# *Babesia canis* and *Anaplasma phagocytophilum* PCR positivity in dogs during the colder half of the year and winter months

**DOI:** 10.1016/j.crpvbd.2026.100455

**Published:** 2026-10-31

**Authors:** Ingo Schäfer, Andreas Moritz, Elisabeth Müller, Christina Strube

**Affiliations:** a Laboklin GmbH & Co. KG, Steubenstrasse 4, 97688, Bad Kissingen, Bavaria, Germany; b Small Animal Clinic – Internal Medicine, Department of Veterinary Medicine, Justus-Liebig-University Giessen, Frankfurter Strasse 114, 35392, Giessen, Hesse, Germany; c Institute for Parasitology, Centre for Infection Medicine, University of Veterinary Medicine Hannover, Buenteweg 17, Haus 217, 30559, Hannover, Lower Saxony, Germany

**Keywords:** Babesiosis, Canine, Climate change, Granulocytic anaplasmosis, Piroplasms, Ticks, Vector-borne disease

## Abstract

*Babesia canis* (vector: *Dermacentor reticulatus*) and *Anaplasma phagocytophilum* (vector: *Ixodes ricinus*) are tick-borne pathogens causing babesiosis and granulocytic anaplasmosis, respectively. The study aimed to demonstrate the rising significance of PCR positivity in dogs in Germany in the colder period (October-March) due to climate change. The database of the laboratory Laboklin was retrospectively screened for *B. canis* and *A. phagocytophilum* PCR results in dogs in Germany during 2008–2025. In cases of multiple testing in a dog, only the first or the first positive result was included. Multiple logistic regression analysis was performed with calculation of odds ratios. A total of 2290 out of 33,542 dogs (6.8%) tested positive for *B. canis* and 2499 out of 50,210 (5.0%) tested positive for *A. phagocytophilum*. Comparing the cold to the warm half of the year, significant differences were demonstrated for *B. canis* (cold: 1282/14,955, 8.6%; warm: 1008/18,587, 5.4%; *P* < 0.001) and *A. phagocytophilum* (cold: 377/20,967, 1.3%; warm: 2222/29,243, 7.6%; *P* < 0.001). *Babesia canis* PCR positivity increased in colder periods (OR = 1.121, *P* = 0.042), including a year-round trend, but decreased for *A. phagocytophilum* (OR = 0.328, *P* < 0.001). This is explainable by vector ecology, with *D. reticulatus* being more cold-tolerant compared to *I. ricinus*. Nevertheless, both pathogens were detected in the cold season, including the classical winter months, highlighting the importance of year-round tick control.

## Highlights

• Retrospective study on diagnostic samples from dogs in Germany during 2008–2025.
• *Babesia canis* PCR-positivity: 8.6% in the cold half of the year, 4.7% in the winter.
• *Anaplasma phagocytophilum* PCR-positivity: 1.3% in the cold half of the year, 1.0% in the winter.
• Infections with *B. canis* increased significantly in the cold season.
• Protection of dogs *via* year-round tick control is essential.

## Graphical abstract

**Figure:**
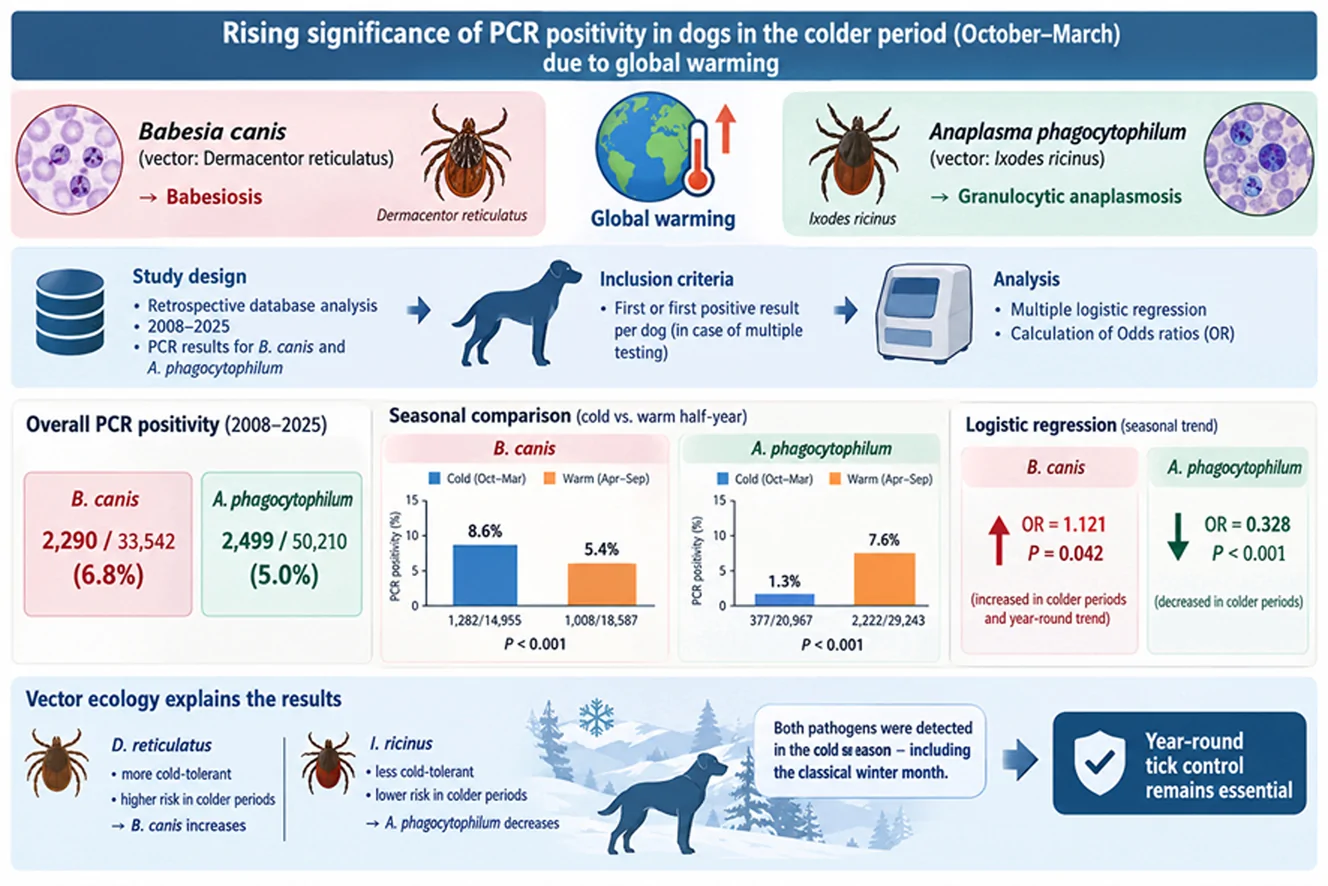

## 1 Introduction

*Babesia canis*, transmitted by the meadow or ornate dog tick *Dermacentor reticulatus* (Gray et al., 2019), and *Anaplasma phagocytophilum*, transmitted by the castor bean or sheep tick *Ixodes ricinus* (Dumler et al., 2001), cause canine babesiosis and granulocytic anaplasmosis in dogs, respectively. Individual tick species distribution is based on their preferred habitats, climate conditions, and presence of host species. *Dermacentor reticulatus*, which is distributed throughout Europe, prefers open landscapes, and infestations in dogs are increasing (Drehmann et al., 2020; Springer et al., 2022), correlating with the spread of the vector tick due to climate change, pet importation, travel, and changes in land use (Springer et al., 2022, 2024). Simultaneously, autochthonous *B. canis* infections have increased in recent years in Germany, especially in highly endemic areas such as Berlin and Brandenburg, Hesse, the Ruhr area, Saxony, and Saxony-Anhalt (Seibert et al., 2022; Weingart et al., 2023; Von Hohnhorst et al., 2025b; Eisenecker et al., 2026). *Dermacentor reticulatus* usually shows a bimodal seasonal activity pattern, with adult ticks being primarily active in colder periods of the year, including peak questing periods in early spring and autumn (Knülle and Dautel, 1997). Adult ticks may become active at air temperatures as low as 0.7 °C and soil temperatures of −0.2 °C, with higher activity and survival from 3.8 °C (Hubalek et al., 2003; Zajac et al., 2020). Due to milder winters in times of climate change, questing adult *D. reticulatus* ticks were recognized in the winter months in several European countries such as Poland, Hungary and Germany (Bartosik et al., 2011; Buczek et al., 2014, 2025; Probst et al., 2023b).

*Ixodes ricinus* ticks are common, especially in central and northern European countries, prefer moderate temperature and high humidity and show a diapause if day length decreases (Gray et al., 2016). In general, *I. ricinus* infestations peak in spring and early summer (Hoch et al., 2024; Marques Alves et al., 2026), with cases of acute granulocytic anaplasmosis peaking in summer (Schäfer et al., 2023b). *Ixodes ricinus* ticks are described to become active when the soil temperature rises above 5–7 °C (Pretzmann et al., 1964). Again, the mild winters due to climate change have led to observations of winter activity of *I. ricinus* (Probst et al., 2023b), which might promote infections with *A. phagocytophilum* during the colder half of the year.

The present study aims to assess patterns of PCR positivity for *B. canis* and *A. phagocytophilum* in dogs in Germany during the colder months due to climate change. Therefore, the database of a commercial German diagnostic laboratory was retrospectively screened for PCR test results between 2008 and 2025 submitted for routine diagnostic testing by veterinarians, focusing on the colder half of the year and winter months.

## 2 Materials and methods

### 2.1 Study design

The database of the laboratory Laboklin (Bad Kissingen, Germany) was retrospectively screened for *B. canis* and *A. phagocytophilum* PCR results in dogs in Germany between January 2008 and December 2025 to analyze infections in the cold half of the year (October-March) and the calendar winter months (December-February). Two study groups were examined, one for *A. phagocytophilum* and the other for *B. canis*. The signalment of dogs included in the study is summarized in Supplementary Table S1. The regional distribution of samples across the individual German federal states is presented in Supplementary Table S2.

The 2008–2020 data for *B. canis* and *A. phagocytophilum* have been published previously to determine regional and classical seasonal distribution patterns as well as risk factors (Schäfer et al., 2023a, 2023b), and parts of the *B. canis* dataset were additionally used to describe clinicopathological abnormalities and acute phase reactions in dogs with acute *B. canis* infections (Von Hohnhorst et al., 2025a, 2025b; Eisenecker et al., 2026).

### 2.2 PCR analysis

Polymerase chain reactions were performed on EDTA-blood from dogs submitted by German veterinarians for routine diagnostics. Data were separately analyzed for *A. phagocytophilum* and *B. canis*. In case of multiple PCR testing for *B. canis* and/or *A. phagocytophilum* in a single dog from 2008 to 2025, only the first or the first positive PCR result was included in the study. *Babesia canis* infections were identified by a piroplasm PCR based on Olmeda et al. (1997) targeting the 18S rRNA gene, and followed by Sanger sequencing in case of a positive test result. For detection of *A. phagocytophilum* infections, a TaqMan real-time PCR targeting the 60-kDa heat shock protein (HSP60) was performed. Details of the PCR conditions have been described in detail previously (Schäfer et al., 2023b).

### 2.3 Statistical analyses

A cold-to-warm ratio was calculated for the individual study years and timeframes for *B. canis* and *A. phagocytophilum* infections by dividing the respective percentage of dogs testing positive by the respective percentage of dogs testing negative. Statistical analyses were conducted using SPSS (v.30.0, IBM), with significance defined as *P* ≤ 0.05. Ninety-five percent confidence intervals (CI) were calculated by the Wilson method, which included continuity correction. Besides individual years, the study’s timeframe was divided into four groups: 2008–2012 (I); 2013–2017 (II); 2018–2022 (III); and 2023-2025 (IV). Differences between these timeframes were assessed by Kruskal-Wallis tests, applying Bonferroni correction, or Mann-Whitney-U testing. Multiple logistic regression was used to compare infections in colder to warmer periods in the individual years and timeframes. Odds ratios (OR) including 95% CI were reported. All figures were generated using GraphPad Prism v.10.5.0 (774) for Windows.

## 3 Results

A total of 68,175 dogs were included in the study.

### 3.1 *Babesia canis*

In total, 2290 of 33,542 dogs (6.8%) tested positive for *B. canis* by PCR and sequencing, with peaks in September (11.5%), October (17.9%) and March (8.5%) (Table 1, Fig. 1A). Winter accounted for a rate of 4.7%, while the rates for the other seasons ranged from 2.7% to 12.8% (Fig. 1A). Notably, PCR positivity was more often recognized in the colder (8.6%) compared to the warmer half of the year (5.4%) (Mann-Whitney-U test, *U* = 143361239.50, *Z* = 11.367, *P* < 0.001) (Table 1, Table 2). Dogs had 61.1% (95% CI: 47.8–75.7%) higher odds of testing positive in the colder period compared to the warmer period (*P* < 0.001).

**Table 1:** *Babesia canis* and *Anaplasma phagocytophilum* PCR positivity in dogs during the cold and warm halves of the years 2008–2025. The PCR positivity (in %) is given with 95% confidence intervals in parentheses, followed by the number of positive samples/total number of samples tested in square brackets.

PCR positivity: Cold half of the year (October-March)			PCR positivity: Warm half of the year (April-September)		
Month	*B*. *canis*	*A*. *phagocytophilum*	Month	*B*. *canis*	*A*. *phagocytophilum*
October	17.9 (16.6–19.2) [570/3193]	2.1 (1.7–2.6) [91/4273]	April	8.5 (7.5–9.5) [253/2990]	4.1 (3.6–4.8) [172/4173]
November	8.9 (7.9–10.0) [248/2795]	1.4 (1.1–1.9) [55/3811]	May	4.6 (3.9–5.4) [148/3228]	8.4 (7.6–9.2) [411/4918]
December	4.0 (3.3–4.9) [88/2204]	1.2 (0.9–1.7) [38/3106]	June	3.9 (3.3–4.6) [133/3418]	12.3 (11.5–13.2) [690/5595]
January	4.4 (3.6–5.4) [96/2174]	0.7 (0.4–1.3) [10/1374]	July	1.8 (1.4–2.3) [56/3143]	10.1 (9.3–10.9) [548/5420]
February	5.6 (4.7–6.7) [121/2148]	0.4 (0.2–0.7) [12/2949]	August	2.4 (1.9–3.1) [68/2777]	5.4 (4.8–6.1) [250/4596]
March	6.5 (5.6–7.6) [159/2441]	1.1 (0.8–1.5) [39/3580]	September	11.5 (10.5–12.7) [350/3031]	3.3 (2.8–3.9) [151/4541]
Total	8.6 (8.1–9.0) [1282/14,955]	1.3 (0.2–2.0) [377/20,967]	Total	5.4 (5.1–5.8) [1008/18,587]	7.6 (7.3–7.9) [2222/29,243]

**Fig. 1:**
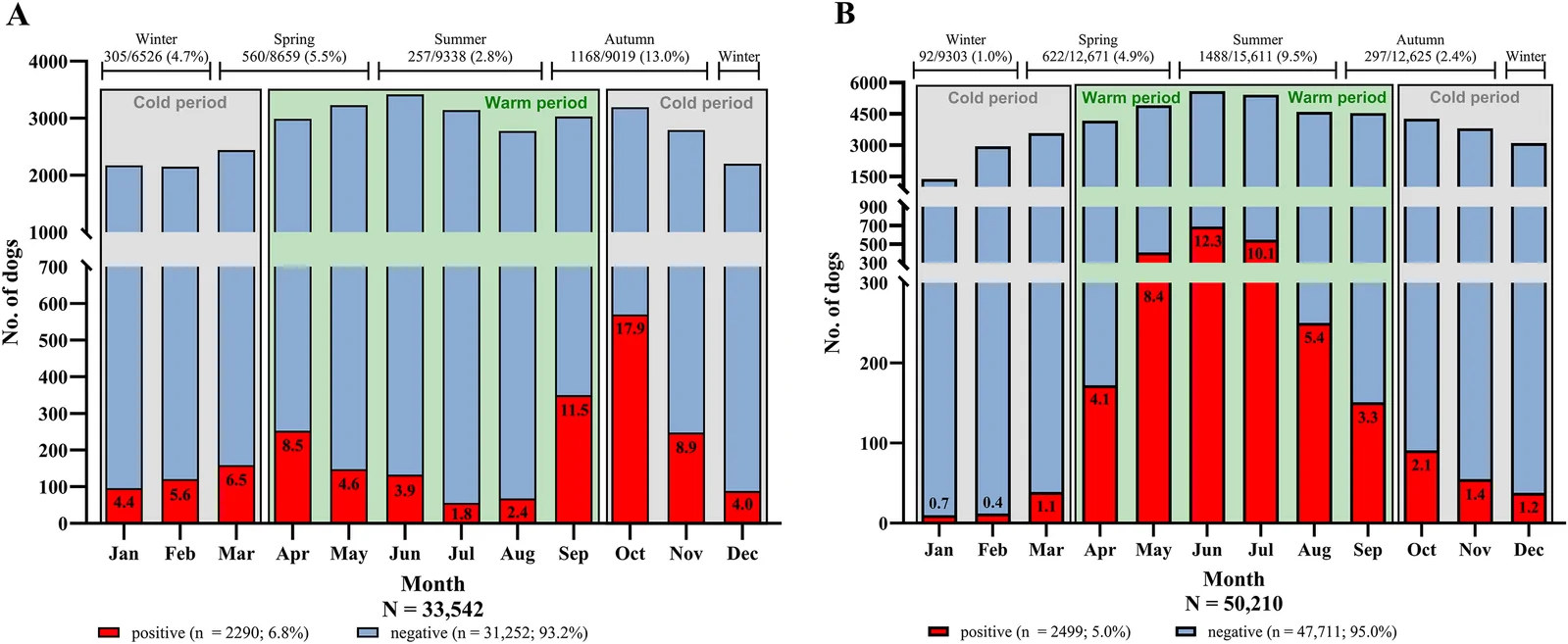
Overview of dogs testing positive for *Babesia canis* (**A**) and *Anaplasma phagocytophilum* (**B**) by PCR during the colder months and winter months between 2008 and 2025. The numbers in the bars indicate the respective monthly percentage of positive dogs.

**Table 2:** *Babesia canis* and *Anaplasma phagocytophilum* PCR positivity in dogs during the cold and warm halves of the years 2008–2025. The PCR positivity (in %) is given with 95% confidence intervals in parentheses, followed by the number of positive samples/total number of samples tested in square brackets. Timeframes: I (2008–2012); II (2013–2017); III (2018–2022); IV (2023–2025).

Timeframe/Year	PCR positivity: Cold half of the year (October-March)		PCR positivity: Warm half of the year (April-September)		PCR positivity ratio cold-to-warm half of the year	
	*B. canis*	*A. phagocytophilum*	*B. canis*	*A. phagocytophilum*	*B. canis*	*A. phagocytophilum*
**Timeframe I**	2.9 (2.2–3.7) [51/1783]	1.5 (1.1–2.1) [37/2396]	2.9 (2.3–3.7) [67/2294]	6.2 (5.4–7.0) [211/3430]	1.00	0.24
2008	1.2 (0.4–3.4) [3/257]	3.5 (2.0–6.0) [12/345]	2.7 (1.5–4.8) [10/375]	5.5 (3.9–7.9) [28/505]	0.44	0.64
2009	2.7 (1.4–5.2) [8/301]	1.5 (0.7–3.0) [7/479]	3.4 (2.0–5.5) [15/446]	6.4 (4.9–8.5) [46/715]	0.79	0.23
2010	3.8 (2.2–6.4) [12/320]	1.0 (0.4–2.4) [5/489]	3.1 (1.8–5.1) [14/456]	4.9 (3.6–6.7) [37/756]	1.22	0.20
2011	2.5 (1.4–4.3) [12/482]	1.6 (0.8–3.2) [8/491]	3.0 (1.8–5.0) [14/465]	6.8 (5.2–8.9) [47/691]	0.83	0.24
2012	3.8 (2.3–6.1) [16/423]	0.8 (0.4–2.0) [5/592]	2.5 (1.5–4.2) [14/552]	6.9 (5.4–9.0) [53/763]	1.52	0.12
**Timeframe II**	3.3 (2.8–4.0) [120/3598]	1.6 (1.3–2.0) [71/4440]	3.1 (2.6–3.6) [148/4766]	7.3 (6.7–8.0) [450/6148]	1.06	0.22
2013	3.7 (2.3–5.7) [18/490]	1.4 (0.7–2.6) [9/655]	3.7 (2.5–5.5) [23/624]	4.6 (3.3–6.3) [37/806]	1.00	0.30
2014	3.5 (2.3–5.2) [22/631]	1.7 (1.0–2.9) [14/810]	2.6 (1.7–4.0) [20/759]	6.4 (5.3–8.3) [70/1049]	1.35	0.27
2015	3.2 (2.1–4.8) [22/694]	1.7 (1.0–2.8) [14/838]	3.8 (2.7–5.3) [32/846]	8.2 (6.7–10.0) [86/1049]	0.84	0.21
2016	2.5 (1.7–3.9) [21/825]	0.8 (0.4–1.6) [8/955]	3.4 (2.5–4.6) [40/1179]	8.2 (6.8–9.7) [116/1423]	0.74	0.10
2017	3.9 (2.8–5.3) [37/958]	2.2 (1.5–3.2) [26/1182]	2.4 (1.7–3.4) [33/1358]	8.0 (6.8–9.3) [141/1773]	1.63	0.28
**Timeframe III**	6.0 (5.4–6.7) [305/5045]	1.3 (1.1–1.6) [110/8274]	3.2 (2.8–3.7) [204/6285]	7.3 (6.8–7.8) [829/11,354]	1.88	0.18
2018	2.4 (1.7–3.5) [27/1111]	1.4 (0.9–2.2) [19/1339]	2.4 (1.7–3.3) [34/1420]	7.6 (6.5–8.9) [138/1821]	1.00	0.18
2019	5.3 (4.2–6.7) [62/1169]	1.6 (1.1–2.4) [24/1510]	2.9 (2.2–4.0) [41/1397]	8.0 (6.9–9.2) [166/2088]	1.83	0.20
2020	4.8 (3.8–6.1) [61/1267]	2.0 (1.5–2.8) [36/1785]	2.6 (1.9–3.5) [41/1570]	7.5 (6.5–8.6) [180/2411]	1.84	0.27
2021	8.8 (7.0–11.1) [64/727]	0.9 (0.6–1.4) [18/2061]	4.0 (2.9–5.3) [42/1061]	5.9 (5.1–6.9) [167/2821]	2.20	0.15
2022	11.8 (9.7–14.3) [91/771]	0.8 (0.5–1.4) [13/1579]	5.5 (4.1–7.3) [46/837]	8.0 (7.0–9.3) [178/2213]	2.15	0.10
**Timeframe IV**	17.8 (16.7–18.9) [806/4529]	1.0 (0.8–1.3) [59/5857]	11.2 (10.4–12.1) [589/5242]	8.8 (8.2–9.4) [732/8311]	1.59	0.11
2023	13.8 (11.7–16.2) [124/901]	0.8 (0.4–1.3) [13/1709]	8.0 (6.4–9.8) [77/968]	8.7 (7.7–10.0) [206/2356]	1.73	0.09
2024	21.7 (19.1–24.5) [194/896]	1.3 (0.9–2.0) [26/1928]	12.2 (10.3–14.5) [114/935]	8.9 (7.9–10.0) [241/2706]	1.78	0.15
2025	17.9 (16.5–19.3) [488/2732]	0.9 (0.6–1.4) [20/2220]	11.9 (10.9–13.1) [398/3339]	8.8 (7.8–9.8) [285/3249]	1.50	0.10
Total	8.6 (8.1–9.0) [1282/14,955]	1.3 (1.2–1.5) [277/20,967]	5.4 (5.1–5.8) [1008/18,587]	7.6 (7.3–7.9) [2222/29,243]	1.59	0.17

Highest percentages of positive dogs were observed in the most recent timeframe IV (14.3%), with a significant rise compared to timeframe I (2.9%), timeframe II (3.2%), and timeframe III (4.5%), respectively (all *P* < 0.001). Significant differences were also detected comparing timeframe III *vs* II (*P* = 0.002) and I (*P* = 0.003), but not for timeframe II to timeframe I (*P* = 1.000).

Across all study years (Fig. 2A), the cold-to-warm ratio was 1.59, and in each timeframe it was ≥ 1.00, i.e. 1.00 in timeframe I, 1.06 in timeframe II, 1.88 in timeframe III, and 1.59 in timeframe IV (Table 2). In the cold half of the year, the comparison of all timeframes demonstrated a significant rise in percentages from timeframe I to IV (I: 2.9; II: 3.3; III: 6.0; IV: 17.8%, *P* < 0.001), except for comparing timeframes I and II (*P* = 1.000). In the warm half of the year, significantly higher percentages were recognized in timeframe IV (11.2%) compared to I (2.9%), II (3.1%), and III (3.2%) (*P* < 0.001, respectively) (Table 2, Table 3).

**Fig. 2:**
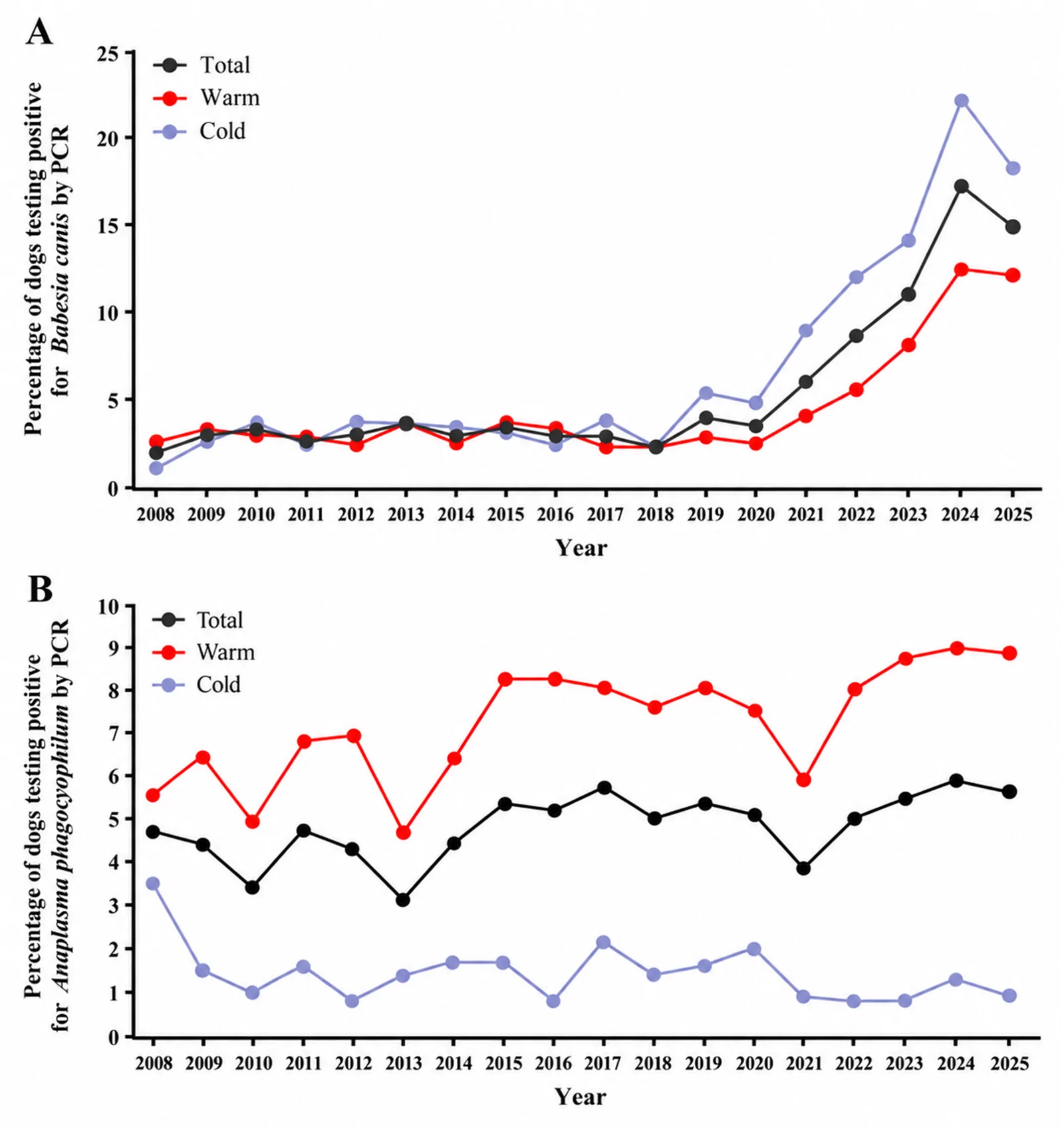
Percentage of dogs testing positive for *Babesia canis* (**A**) and *Anaplasma phagocytophilum* (**B**) by PCR from 2008 to 2025. The warm period was defined as April-September; the cold period was defined as October-March.

**Table 3:** Statistical comparisons of *Babesia canis* and *Anaplasma phagocytophilum* PCR positivity in dogs during the cold and warm halves of the years 2008–2025. Timeframes: I (2008–2012); II (2013–2017); III (2018–2022); IV (2023–2025).

Timeframe	PCR positivity: Whole year		PCR positivity: Cold half of the year (October-March)		PCR positivity: Warm half of the year (April-September)	
	*B. canis*	*A. phagocytophilum*	*B. canis*	*A. phagocytophilum*	*B. canis*	*A. phagocytophilum*
	*P*-value	*P*-value	*P*-value	*P*-value	*P*-value	*P*-value
I *vs* II	1.000	0.625	1.000	0.591	1.000	0.156
I *vs* III	0.003*	0.368	<0.001*	0.315	1.000	0.232
I *vs* IV	<0.001*	0.001*	<0.001*	0.055	<0.001*	<0.001*
II *vs* III	0.002*	1.000	<0.001*	1.000	1.000	1.000
II *vs* IV	<0.001*	0.005*	<0.001*	1.000	<0.001*	<0.001*
III *vs* IV	<0.001*	0.106	<0.001*	1.000	<0.001*	0.005*

*Note*: Kruskal-Wallis test including Bonferroni *post-hoc* correction; *P ≤ 0.05 stated as statistically significant.

### 3.2 *Anaplasma phagocytophilum*

Overall, 2499 of 50,210 dogs (5.0%) tested positive for *A. phagocytophilum* with peaks in May (8.4%), June (12.3%), and July (10.1%) (Table 1, Fig. 1B). The percentage of PCR positivity in winter of dogs with *A. phagocytophilum* was quite low with 1.0%, in the other seasons the values ranged from 2.4% to 9.5% (Fig. 1B). PCR positivity was predominantly observed during the warm half of the year (7.6%), compared to the colder half (1.3%) (Mann-Whitney-U test, *U* = 287324809.00, *Z* = −32.897, *P* < 0.001) (Table 1, Table 2). Dogs had 614.1% (95% CI: 541.3–696.7%) higher odds of testing positive in the warmer period compared with the colder period (*P* < 0.001).

Evaluating the percentages of PCR-positive dogs over the four timeframes (Fig. 2B) demonstrated highest percentages in timeframe IV (5.6%), with significant differences compared to timeframes I (4.3%, *P* = 0.001) and III (4.8%, *P* = 0.005). No significant differences were detected between timeframe I and II (4.9%) (*P* = 0.368), I and III (*P* = 0.625), and II and III (*P* = 1.000), as well as II and IV (*P* = 0.106).

The cold-to-warm ratio across all study years was 0.17. It decreased in each timeframe, i.e. from 0.24 in timeframe I to 0.22 in timeframe II, 0.18 in timeframe III, and 0.11 in timeframe IV (Table 2). Significant differences were detected in the warm half of the year comparing timeframe I (6.2%) *vs* IV (8.8%) (*P* < 0.001), II (7.3%) *vs* IV (*P* < 0.001), and III (7.3%) *vs* IV (*P* = 0.005). No significant impact of timeframes was demonstrated in the cold half of the year (Table 2, Table 3).

### 3.3 Co-infections

Co-infections with both pathogens were identified in 19 of 15,560 dogs (0.1%) with parallel testing for both pathogens. Of these 19 dogs, 12 (63.2%) were tested in 2025, three (15.8%) in 2024, and one each (5.3%) in 2009, 2011, 2015, and 2019. Most co-infections were detected in June (*n* = 9; 47.4%), followed by May (*n* = 4; 21.1%), April and September (*n* = 2, each; 10.5%) as well as February and July (*n* = 1, each; 5.3%).

## 4 Discussion

Climate change in terms of milder cold periods or winters, respectively, will lead to a year-round increased risk of infection with tick-borne pathogens in humans and animals (Probst et al., 2023b). To gain insight into *B. canis* and *A. phagocytophilum* PCR-positivity in dogs during the cold half of the year, we analyzed diagnostic PCR results over a 13-year period. Both pathogens showed trends toward rising significance in dogs in Germany, which has been especially demonstrated for *B. canis* in other studies (Seibert et al., 2022; Schäfer et al., 2023a; Weingart et al., 2023). Although diagnostic laboratory results often overestimate the actual prevalence because samples are frequently submitted due to clinical suspicion, data trends are nonetheless useful for identifying potential shifts, such as those caused by climate change. Additionally, the persistence of parasite DNA must be considered in the case of *B. canis* but seems unlikely in the case of *A. phagocytophilum* due to the acute onset of granulocytic anaplasmosis. Clinical data were missing in the dogs included in our study, and therefore individual cases of prolonged parasitemia may have influenced the cold-to-warm ratio especially regarding *B. canis*.

Interestingly, PCR positivity for *B. canis* was more often recognized in the cold compared to the warm periods of the year, while it was the other way round for *A. phagocytophilum*. This is most likely linked to the activity patterns of the transmitting tick vectors. *Dermacentor reticulatus* tolerates colder temperatures with higher activity already from an air temperature of 3.8 °C (Hubalek et al., 2003), while *I. ricinus* ticks are described to be activated at 5–7 °C (Pretzmann et al., 1964). These tick activity patterns seem to be reflected in the percentages of infections with both pathogens; however, no closer correlation between environmental factors or the distribution of tick vectors has been analyzed and is worth addressing in further studies. Nevertheless, PCR positivity for both *B. canis* and *A. phagocytophilum* has been detected during the winter months, i.e. December-February, at rates of 4.7% and 1.0%, respectively, implying that both vector tick species are meanwhile active in a season previously considered to be (largely) “questing tick-free” in Germany (Dautel et al., 2008; Gray et al., 2009; Bartosik et al., 2011; Probst et al., 2023a). Interestingly, the PCR positivity ratio between the cold and warm half of the year increased markedly for *B. canis* as of timeframe III, i.e. during 2018–2022, while it decreased notably for *A. phagocytophilum* as of timeframe IV, i.e. during the last three study years (2023–2025). Regarding *B. canis*, this pattern was driven by a prominent increase in prevalence during the cooler half of the year. Besides enhanced *D. reticulatus* activity due to milder winters, this might be linked to the spread of this tick species throughout Germany (Drehmann et al., 2020; Springer et al., 2022), followed by rising autochthonous *B. canis* infections instead of the previous predominance of imported cases (Schäfer et al., 2023a; Von Hohnhorst et al., 2025b). For *A. phagocytophilum*, the decreased ratio was mainly due to an increased percentage in the warm half of the year; however, follow-up data are necessary to elicit whether this is a long-term trend or merely a transient event.

The rising occurrence of co-infections with *B. canis* and *A. phagocytophilum* in the last two years of our study period seems to reflect the increasing overlap in activity patterns of *I. ricinus* and *D. reticulatus* ticks in Germany or a rising awareness among veterinarians. Of note, babesiosis due to *B. canis* and granulocytic anaplasmosis share similar clinicopathological abnormalities like lethargy, fever, inappetence, and thrombocytopenia as the most common clinicopathological abnormalities (Chirek et al., 2018; Weingart et al., 2023; von Hohnhorst et al., 2025b; Eisenecker et al., 2026). Overall, the study results showing frequent detection of canine babesiosis, but also granulocytic anaplasmosis, during the colder half of the year, including the winter months, highlight the importance of effective year-round tick control in dogs with licensed acaricides.

## 5 Conclusions

*Babesia canis* infections increased in colder periods, including a trend for year-round infections, but decreased for *A. phagocytophilum*. This can be explained by vector ecology, with *D. reticulatus* being more cold-tolerant. Rising numbers of co-infections with *B. canis* and *A. phagocytophilum* were detected in 2024 and 2025, which might reflect the increasing overlap in activity patterns of *I. ricinus* and *D. reticulatus* ticks in Germany due to climate change and/or a rising awareness among veterinarians. Both pathogens were also detected in the cold season including the classical winter month, highlighting the importance of year-round tick control with licensed acaricides.

## Ethical approval

No ethical approval was required as all analyses were performed retrospectively on samples submitted for routine diagnostics by veterinarians in Germany. Data were anonymized for statistical evaluation. The secondary use of anonymized diagnostic data for research purposes is covered by institutional policy, and individual owner consent was not required.

## CRediT authorship contribution statement

**Ingo Schäfer:** Conceptualization, Investigation, Visualization, Writing - original draft, Writing - review & editing. **Andreas Moritz:** Supervision, Writing - review & editing. **Elisabeth Müller:** Writing - review & editing. **Christina Strube:** Conceptualization, Supervision, Writing - review & editing.

## Data availability

All data generated or analyzed during this study are included in this published article and its supplementary file.

## Funding

This research did not receive any specific grant from funding agencies in the public, commercial, or not-for-profit sectors.

## Declaration of competing interests

The authors declare the following financial interests/personal relationships which may be considered as potential competing interests: Ingo Schäfer is an employee, and Elisabeth Müller is the CEO of Laboklin GmbH & Co. KG; Andreas Moritz, Christina Strube, and Ingo Schäfer have repeatedly lectured for and/or acted as consultants for diagnostic and (veterinary) pharmaceutical companies; Andreas Moritz and Christina Strube have previous and ongoing research collaborations with various diagnostic and (veterinary) pharmaceutical companies.
