# Supplementary material for: Babesia canis and Anaplasma phagocytophilum PCR positivity in dogs during the colder half of the year and winter months
Source: Curr Res Parasitol Vector Borne Dis. 2026 Oct 31;10:100455. doi: 10.1016/j.crpvbd.2026.100455 (PMC13756335; doi:10.1016/j.crpvbd.2026.100455)
Supplement: Supplementary file 1 [file mmc1.pdf]

Supplementary Table S1. Signalment of dogs testing for *Anaplasma phagocytophilum* and/or *Babesia canis* by polymerase chain reaction on EDTA blood in Germany from 2008-2025 (*n*/*N* total (%, [95% CI lower limit;95% CI upper limit])).

		***Anaplasma phagocytophilum* PCR total**	***Anaplasma phagocytophilum* PCR-positive**	***Anaplasma phagocytophilum* PCR-negative**	***Babesia canis* PCR total**	***Babesia canis* PCR-positive**	***Babesia canis* PCR-negative**
**Sex**	Male intact	15,165/47,507 (31.9 [31.5;32.3])	797/2365 (33.7 [31.8;35.6])	14,368/45,142 (31.8 [31.4;32.3])	8596/31,197 (27.6 [27.6;28.1])	761/2129 (35.7 [33.7;37.8])	7835/29,068 (27.0 [26.5;27.5])
	Male castrated	9387/47,507 (19.8 [19.4;20.1])	475/2365 (20.1 [18.5;21.8])	8912/45,142 (19.7 [19.4;20.1])	6722/31,197 (21.5 [21.1;22.0])	369/2129 (17.3 [15.8;19.0])	6353/29,068 (21.9 [21.4;22.3])
	Female	11,983/47,507 (25.2 [24.8;25.6])	525/2365 (22.2 [20.6;23.9])	11,458/45,142 (25.4 [25.0;25.8])	7503/31,197 (24.1 [23.6;24.5])	601/2129 (28.2 [26.4;30.2])	6902/29,068 (23.7 [23.3;24.2])
	Female spayed	10,972/47,507 (23.1 [22.7;23.5])	568/2365 (24.0 [22.3;25.8])	10,404/45,142 (23.0 [22.7;23.4])	8376/31.197 (26.8 [26.4;27.3])	398/2129 (18.7 [17.1;20.4])	7978/29,068 (27.4 [26.9;28.0])
	Unknown	2703/50,210 (5.4 [5.2;5.6])	134/2499 (5.4 [4.5;6.3])	2569/47,711 (5.4 [5.2;5.6])	2345/33,542 (7.0 [6.7;7.3])	161/2290 (7.0 [6.1;8.2])	2184/31,252 (7.0 [6.7;7.3])
**Age (in years)**	Unknown	3734/50,210 (7.4 [7.2;7.7])	174/2499 (7.0 [6.0;8.0])	3560/47,711 (7.5 [7.2,7.7])	4441/33,542 (13.2 [12.9;13.6])	353/2290 (15.4 [14.0;17.0])	4088/31,252 (13.1 [12.7;13.5])
	Available	46,476/50,210 (92.6 [92.3;92.8])	2325/2499 (93.0, [92.0;94.0]	44,151/47,711 (92.5 [92.3;92.8]]	29,101/33,542 (86.8 [86.4;87.1])	1937/2290 (84.6 [83.1;86.0])	27,164/31,252 (86.9 [86.5;87.3])
	Mean (years)	6.8	7.1	6.7	6.5	5.8	6.6
	Median (years)	7.0	7.0	7.0	6.0	5.0	6.0
	Standard deviation (years)	3.9	3.9	3.9	3.9	3.7	3.9
	Minimum (months)	3	3	3	2	3	2
	Maximum (years)	19	19	19	18	18	18
**Breed**	Pure bred	32,677/47,700 (68.5 [68.1;68.9])	1744/2391 (72.9 [71.1;74.7])	30,933/45,309 (68.3 [67.8;68.7])	17,182/31,055 (55.3 [54.8;55.9])	1272/2137 (59.5 [57.4;61.6])	15,910/28,918 (55.0 [54.4;55.6])
	Mixed bred	15,023/47,700 (31.5 [31.1;31.9])	647/2391 (27.1 [25.3;28.9])	14,376/45,309 (31.7 [31.3;32.2])	13,873/31,055 (44.7 [44.1;45.2])	865/2137 (40.5 [38.4;42.6])	13,008/28,918 (45.0 [44.4;45.6])
	Unknown	2510/50,210 (5.0 [4.8;5.2])	108/2499 (4.3 [3.6;5.2])	2402/47,711 (5.0 [4.8;5.2])	2487/33,542 (7.4 [7.1;7.7])	153/2290 (6.6 [5.7;7.8])	2334/31,252 (7.5 [7.2;7.8])
**Total**	-	**50,210**	**2,499**	**47,711**	**33,542**	**2,290**	**31,252**

Abbreviations: CI, confidence interval; EDTA, ethylenediamine tetra-acetic acid; PCR, polymerase chain reaction.

Supplementary Table S2. Dogs testing for *Anaplasma phagocytophilum* and/or *Babesia canis* by polymerase chain reaction on EDTA blood in different German federal states from 2008-2025 (*n* positive/*N* total (% [95% CI lower limit;95% CI upper limit]).

Federal state	*Anaplasma phagocytophilum* PCR	*Babesia canis* PCR	Regionality	*Anaplasma phagocytophilum* PCR	*Babesia canis* PCR
**Bavaria**	244/4573 (5.3 [4.7;6.0])	115/2333 (4.9)	**South**	768/18,298 (4.2 [3.9;4.5])	637/12,089 (5.3 [4.9;5.7])
**Baden-Wuerttemberg**	234/5411 (4.3 [3.8;4.9])	180/3418 (5.3)			
**Hesse**	140/4044 (3.5 [2.9;4.1])	180/2701 (6.7)			
**Rhineland Palatinate**	110/3206 (3.4 [2.9;4.1])	80/2840 (2.8)			
**Saarland**	40/1064 (3.8 [2.8;5.1])	82/797 (10.3)			
**Lower Saxony/Bremen**	593/9065 (6.5 [6.1;7.1])	380/5012 (7.6)	**North**	1731/31,912 (5.4 [5.2;5.7])	1653/21,453 (7.7 [7.3;8.0])
**Saxony-Anhalt**	35/870 (4.0 [2.9;5.5])	171/869 (19.7)			
**Saxony**	117/1791 (6.5 [5.5;7.8])	137/1110 (12.3)			
**Thuringia**	51/793 (6.4 [4.9;8.4])	18/471 (3.8)			
**Brandenburg/Berlin**	411/6886 (6.0 [5.4;6.6])	627/4855 (12.9)			
**Schleswig-Holstein/Hamburg**	57/1188 (4.8 [3.7;6.2])	38/1072 (3.5)			
**Mecklenburg Western Pomerania**	46/890 (5.2 [3.9;6.8])	56/648 (8.6)			
**Total**	**2499/50,210 (5.0 [4.8;5.2])**	**2290/33,542 (6.8 [6.6;7.1])**	**Total**	**2499/50,210 (5.0 [4.8;5.2])**	**2290/33,542 (6.8 [6.6;7.1])**

Abbreviations: CI, confidence interval; EDTA, ethylenediamine tetra-acetic acid; PCR, polymerase chain reaction.
